# Laparoscopic-Assisted Endoscopic Retrograde Cholangiopancreatography (ERCP) Versus Endoscopic Ultrasound-Directed Transgastric ERCP in Patients With Roux-en-Y Gastric Bypass: A Systematic Review and Meta-Analysis

**DOI:** 10.7759/cureus.30196

**Published:** 2022-10-11

**Authors:** Victor L de Oliveira, Diogo Turiani H de Moura, Epifânio S do Monte Júnior, Igor M Proença, Igor B Ribeiro, Sergio A Sánchez-Luna, Pedro Henrique Boraschi V Ribas, Matheus C Hemerly, Wanderley M Bernardo, Eduardo Guimarães H de Moura

**Affiliations:** 1 Gastroenterology, Hospital das Clínicas da Universidade de São Paulo, Sao Paulo, BRA; 2 Gastroenterology, University of Alabama at Birmingham Marnix E. Heersink School of Medicine, Birmingham, USA

**Keywords:** laparoscopy, endoscopic ultrasound (eus), endoscopic retrograde cholangiopancreatography (ercp), roux-en-y gastric bypass (rygb), surgery, endoscopy

## Abstract

Endoscopic retrograde cholangiopancreatography (ERCP) is a therapeutic procedure for skilled endoscopists that can be even more challenging in some situations, including patients' post-Roux-en-y Gastric Bypass (RYGB) surgery. There is still no consensus on whether laparoscopic-assisted ERCP (LA-ERCP) or endoscopic ultrasound (EUS)-directed transgastric ERCP (EDGE) is the most appropriate, safe, and feasible approach in patients with this type of post-surgical anatomy. This systematic review and meta-analysis aimed to examine both approaches' feasibility, efficacy, and safety in this situation.

We searched for electronic databases (MEDLINE, EMBASE, Lilacs, Google Scholar, and Central Cochrane) to identify studies comparing LA-ERCP versus EDGE. Outcomes measured included technical success, adverse events (AEs) and serious AEs, length of stay (LOS), and procedural time. Descriptive data related to the EDGE procedure was also extracted. The risk of bias and the quality of evidence of the enrolled studies were assessed.

Five studies, totalizing 268 patients (176 LA-ERCP and 92 EDGE), were included. There was no statistical difference in technical success and AEs between groups; however, the LOS and procedural times were shorter for the EDGE group. High rates of fistula closure and no weight regain were observed in EDGE. Both methods are feasible and safe techniques to perform ERCP in patients with RYGB anatomy, with comparable technical success and adverse events rate. However, EDGE is associated with shorter LOS and procedural time.

## Introduction and background

Obesity is a public health problem that leads to increasing bariatric and metabolic surgical interventions, including Roux-en-y Gastric Bypass (RYGB) surgery. The abrupt weight loss raises the risk of developing gallstones and, consequently, choledocholithiasis and its complications [1], requiring endoscopic retrograde cholangiopancreatography (ERCP) as a therapy. However, accessing the biliary tree in these patients is challenging, especially by enteroscopy, due to increased difficulty in biliary cannulation, limited devices available for therapy of choledocholithiasis while using an enteroscope, and the need for special devices (due to the narrow diameter and long length of the working channel) [2]. Therefore, alternative techniques have been recently proposed and demonstrated superior results compared to enteroscopy-assisted ERCP (EA-ERCP) [3-5].

Laparoscopy-assisted ERCP (LA-ERCP) was first described in 2002 and consisted of creating a gastrostomy at the excluded stomach followed by the insertion of a conventional duodenoscope through the laparoscopic port. This procedure allows for performing a standard ERCP immediately during intraoperative [6]. The demand for increased coordination between endoscopic and surgical teams and the difficulty of performing consecutive ERCPs by this method are significant limitations of this approach [7,8].

Endoscopic ultrasound (EUS)-directed transgastric ERCP (EDGE) was initially reported in 2014 [9] and is typically performed by creating transluminal access between the gastric pouch or the proximal efferent limb and the gastric remnant using a lumen-apposing metal stent (LAMS) under EUS and fluoroscopic guidance. The risk of fistula persistence after LAMS removal and its associated complications, such as weight regain, especially in fistulas with long-term patency, have always been a concern of this therapeutic modality [8]. 

To better understand and evaluate each approach's feasibility, efficacy, and safety for this challenging population, we conducted this systematic review and meta-analysis. We examined the most recent and best quality of evidence available in the literature to compare EDGE and LA-ERCP outcomes directly.

## Review

Materials and methods

Protocol and Registration

This systematic review and meta-analysis were conducted according to the Cochrane Handbook of Systematic Reviews of Interventions and the Preferred Reporting Items for Systematic Reviews and Meta-analysis (PRISMA) guidelines [10]. The study protocol was registered in the International Prospective Register of Systematic Reviews (PROSPERO) under the file number CRD42021257219 and was approved by the Ethics Committee.

Eligibility Criteria

To provide a better quality of evidence and limit the heterogeneity between study groups, only studies directly comparing EDGE and LA-ERCP in post-RYGB patients were included, either with prospective or retrospective design. All relevant articles (abstracts or full-text manuscripts) were considered for inclusion, regardless of language and year of publication. Exclusion criteria included non-comparative studies (case reports, case series, narrative reviews, editorials), studies in patients with any other surgical altered anatomy, and studies that did not report at least technical success rate and adverse events (AEs) outcomes. When articles with a concern of sample duplication were identified, only the most recent was included. Studies with missing data and failed contact with the author to retrieve data were also excluded.

Search Strategy and Information Sources

Individualized searches of electronic databases (MEDLINE, EMBASE, Lilacs, Google Scholar, and Central Cochrane) and grey literature were conducted from inception through October 10, 2022. The search strategy was the same in all databases as follows: (Gastric Bypass OR Gastrojejunostomy OR Gastrojejunostomies OR Gastroileal Bypass OR Transgastric OR Roux-en-Y) AND (Retrograde Cholangiopancreatography, Endoscopic OR Endoscopic Retrograde Cholangiopancreatographies OR ERCP OR Endoscopic Ultrasound OR Endosonography OR Endosonographies OR Endoscopy, Ultrasonic OR Ultrasonic Endoscopies OR Endoscopic Ultrasonography OR Endoscopic Ultrasonographies). From the initial results, duplicate articles were removed, and then titles and abstracts were assessed for eligibility by two independent investigators. Any disagreements were resolved by consultation with a third reviewer.

Data Collection Process and Measured Outcomes

Studies considered relevant were selected for full-text analysis, and Excel sheets were used to collect the appropriate data and results. Descriptive data extracted included age, sex, the indication of the procedure, and time from RYGB to the procedure. For the EDGE group of patients, specific outcomes and procedural details (weight regain, fistula closure rate, access type, LAMS size, and single/two-stage procedure) were also extracted and registered. However, they could not take a comparative analysis.

The primary outcomes included technical success and AEs to establish an adequate comparison of the feasibility and safety of both procedures. Other secondary outcomes measured were procedural time and length of hospitalization (LOS). Technical success was defined as the capacity to reach the ampulla and perform therapeutic ERCP after accessing the excluded stomach. All the studies analyzed assumed that clinical success was achieved after successful therapeutic ERCP. Thus, we considered only technical success as an outcome of meta-analysis.

The AEs were classified according to the 2010 American Society for Gastrointestinal Endoscopy (ASGE) lexicon for endoscopic AEs [11] or graded according to the Clavien-Dindo classification [12] if surgical complications, with grade III and IV defined as severe. For the serious AEs analysis, both mild and moderate AEs were grouped as non-serious AEs. Secondary outcomes evaluated included the LOS and procedural time.

Risk of Bias in Individual Studies and Quality of Evidence

The risk of bias was assessed by Cochrane's Risk of Bias in Non-randomized Studies of Interventions (ROBINS-I) [13]. The quality of the evidence was assessed using the objective criteria of Grading of Recommendations Assessment, Development, and Evaluation (GRADE) for each outcome using the GRADEpro - Guideline Development Tool software [14].

Data Analysis

The software Review Manager (RevMan), version 5.4 - Cochrane Collaboration Copyright © 2014, was used to create tabular and graphical displays and perform the statistical analysis. Absolute values, means, and standard deviations (SD) were used in the data analysis. If a study provided medians and measures of variance, mathematical formulae were used to estimate means and SD, thus promoting data standardization [15]. We calculated the risk difference (RD) for dichotomous variables using the Mantel-Haenszel test. The mean difference (MD) value was calculated using means and SD by inverse variance test for continuous variables. A confidence interval (CI) of 95% was established for both measures. All calculated p values were two-sided, and p values < 0.05 were considered statistically significant. Heterogeneity was assessed employing the Higgins method (I²) and Chi-Squared test (X²). Values of heterogeneity less than 50% were considered low, and fixed-effects models were applied.

Results

Search Results and Study Characteristics

**Figure 1:**
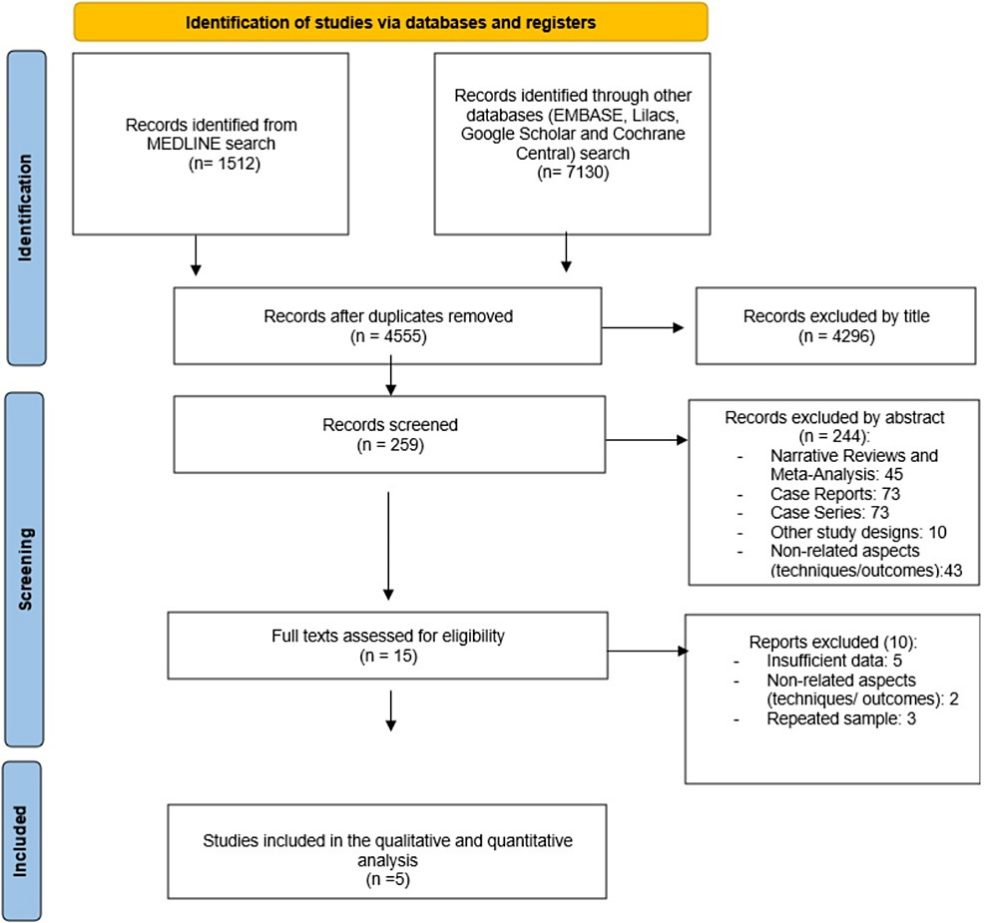
Flow diagram showing the study selection process for meta-analysis.

A total of 8642 studies were identified from the initial search. A total of four retrospective studies [16-19] and one abstract of a retrospective cohort [20] were included in this study, as summarized in Figure 1. These five studies represented a sample of 268 patients, 92 in the EDGE group and 176 in the LA-ERCP group, whose individual characteristics are summarized in Table 1.

**Table 1:** Characteristics of patients and interventions EDGE: Endoscopic Ultrasound-Directed Transgastric ERCP; LA-ERCP: Laparoscopic-Assisted Endoscopic Retrograde Cholangiopancreatography; RYGB: Roux-en-Y Gastric Bypass; NI: Not Informed

Author (year)	Intervention (n)	Study Design	Age, years (variance)	Indication, n (%)	Time from RYGB, years (variance)	Outcomes
Kedia (2019) [16]	EDGE (29)	Retrospective Cohort	56 (35-82)	Biliary: 23 (79%) Pancreatic: 6 (21%)	5.5 (NI)	Technical Success, Adverse Events, Severe Adverse Events, Length of Hospitalization, Procedure Time
	LA-ERCP (43)		55 (33-80)	Biliary: 36 (84%) Pancreatic: 7 (16%)	7.7 (NI)	
Parvataneni (2019) [20]	EDGE (17)	Retrospective Cohort	55.9 (NI)	NI	NI	Technical Success, Adverse Events Procedure Time
	LA-ERCP (59)		55.6 (NI)	NI	NI	
Kroll (2020) [18]	EDGE (2)	Retrospective Cohort	50.5 (49-52)	Biliary: 2 (100%)	5 (0-12)	Technical Success, Adverse Events, Severe Adverse Events, Length of Hospitalization, Procedure Time
	LA-ERCP (14)		45.5 (28-72)	Biliary: 14 (100%)	5 (0-12)	
Kochhar (2020) [17]	EDGE (26)	Retrospective Cohort	60.77 (11.44)	Biliary: 22 (85%) Pancreatic: 4 (15%)	10.7 (NI)	Technical Success, Adverse Events, Severe Adverse Events, Length of Hospitalization, Procedure Time
	LA-ERCP (18)		60.78 (12.67)	Biliary: 16 (89%) Pancreatic: 2 (11%)	10.7 (NI)	
Wang (2021) [19]	EDGE (18)	Retrospective Cohort	59.3 (6.5)	Biliary benign: 14 (78%) Malignance:4 (22%)	13.2 (7.6)	Technical Success, Adverse Events, Severe Adverse Events, Length of Hospitalization
	LA-ERCP (42)		50.6 (15.9)	Biliary: 37 (88%) Pancreatic: 5 (12%)	8.4 (5.2)	

Risk of Bias and Quality of Evidence Assessment

**Figure 2:**
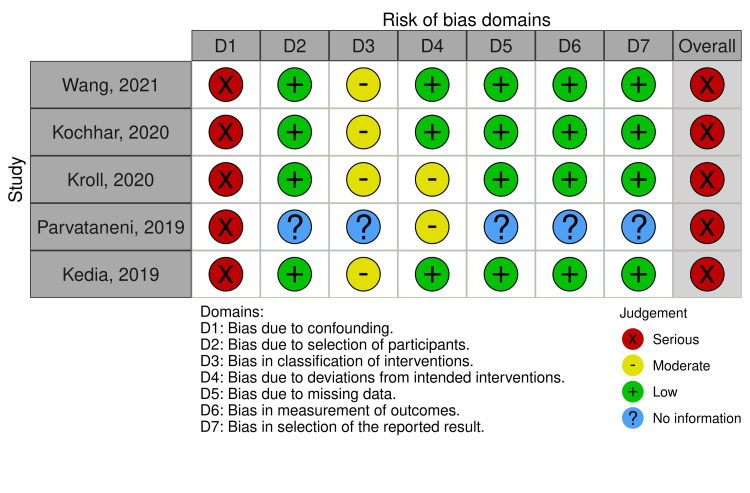
ROBINS-I – Risk of Bias assessment Studies represented in the figure [16-20]

All studies [16-20] presented a severe overall risk of bias (Figure 2). The quality of evidence for each outcome is described in Table 2.

**Table 2:** GRADE analysis for the EDGE vs. LA-ERCP comparison CI: confidence interval; MD: mean difference; RR: risk ratio; LA-ERCP: Laparoscopic-assisted endoscopic retrograde cholangiopancreatography; Explanations a. Confounding Factors were not controlled b. Classification of intervention groups not well-defined c. Large Range of results d. Not all studies clearly stratified adverse events severity e. The outcome does not represent directly the superiority of the technique

Participants (studies) Follow-up	Risk of bias	Inconsistency	Indirectness	Imprecision	Publication bias	Overall certainty of evidence	Study event rates (%)		Relative effect (95% CI)	Anticipated absolute effects	
							With [LA-ERCP]	With [EDGE]		Risk with [LA-ERCP]	Risk difference with [EDGE]
Technical Success											
268 (5 observational studies) [16-20]	serious^a^	not serious	not serious	not serious	none	⨁⨁⨁◯ Moderate	173/176 (98.3%)	90/92 (97.8%)	RR 1.00 (0.95 to 1.05)	983 per 1.000	0 fewer per 1.000 (from 49 fewer to 49 more)
Adverse Events											
268 (5 observational studies) [16-20]	very serious^a,b^	not serious	not serious	serious^c^	none	⨁◯◯◯ Very low	36/176 (20.5%)	12/92 (13.0%)	RR 0.63 (0.33 to 1.20)	205 per 1.000	76 fewer per 1.000 (from 137 fewer to 41 more)
Severe Adverse Events											
192 (4 observational studies) [16-19]	very serious^a,d^	not serious	not serious	not serious	none	⨁⨁◯◯ Low	5/117 (4.3%)	2/75 (2.7%)	RR 0.69 (0.18 to 2.55)	43 per 1.000	13 fewer per 1.000 (from 35 fewer to 66 more)
Length of Hospitalization											
192 (4 observational studies) [16-19]	serious^a^	not serious	not serious	not serious	none	⨁⨁⨁◯ Moderate	117	75	-	The mean length of Hospitalization was 0	MD 1.2 lower (1.86 lower to 0.53 lower)
Procedural Time											
208 (4 observational studies) [16-18, 20]	serious^a^	not serious	serious^e^	not serious	none	⨁⨁◯◯ Low	134	74	-	The mean procedure Time was 0	MD 98.62 lower (113.62 lower to 83.63 lower)

Characteristics and Outcomes of the EDGE Procedure Group

Table 3 shows specific data regarding the pool of patients that underwent the EDGE procedure. Fistula closure - performed through some endoscopic adjuvant techniques in most cases - was reported by four studies [16-19] and was achieved in the majority of patients (85%). Spontaneous fistula closure was achieved in 24% of the cases. Four studies [16,17,19,20] assessed weight change and none of them found significant weight regain after the EDGE procedure. Gastrogastrostomy (GG) was the most common transluminal access to the excluded stomach, followed by jejunogastrostomy (JG). The procedure was performed mainly in two stages, and the 15 mm LAMS was the most commonly employed size stent among studies with available data [16,18].

**Table 3:** Characteristics and Outcomes of EDGE group GG: gastrogastrostomy; JG: jejunogastrostomy; NI: not informed; LAMS: lumen-apposing metal stents.

Author (year)	Patients, n	Weight Change, average, kg	Fistula Closure, n (%)	Access Type, GG/JG (%)	One Stage/Two Stages, n (%)	LAMS Size, 15mm/20mm (%)
Kedia (2019) [16]	29	-3	29 (100%)	NI	3/26 (10%/90%)	29/0 (100%/0%)
Parvataneni (2019) [20]	17	-2.9	NI	NI	17/0 (100%/0%)	NI
Kroll (2020) [18]	2	NI	2 (100%)	0/2 (0%/100%)	0/2 (0%/100%)	NI
Kochhar (2020) [17]	26	-1.4	22 (85%)	22/4 (84.6%/15.4%)	13/13 (50%/50%)	24/2 (92%/8%)
Wang (2021) [19]	18	-2.6	11 (61%)	NI	NI	NI

Meta-analysis

Technical Success

**Figure 3:**
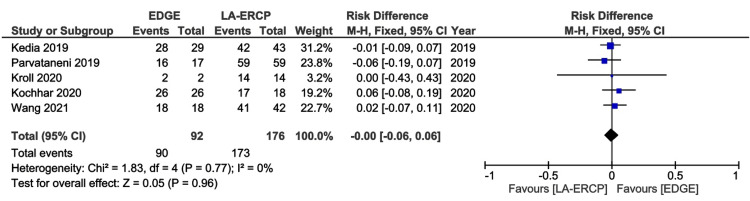
Forest Plot of Technical Success EDGE: Endoscopic Ultrasound-Directed Transgastric ERCP; LA-ERCP: Laparoscopic-Assisted Endoscopic Retrograde Cholangiopancreatography; M-H: Mantel-Haenszel; CI: Confidence interval Studies represented the forest plot [16-20]

All studies reported technical success [16-20]. The rates of technical success were similar, 97.8% and 98.3% in the EDGE and LA-ERCP groups, respectively, with a RD of 0.00 (95% CI − 0.06 to 0.06; I² = 0%; p = 0.96) (Figure 3).

Adverse Events and Serious Adverse Events

**Figure 4:**
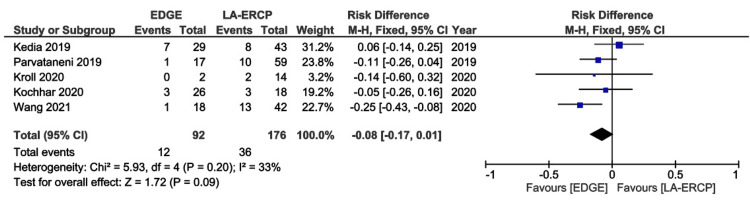
Forest Plot of Adverse Events EDGE: Endoscopic Ultrasound-Directed Transgastric ERCP; LA-ERCP: Laparoscopic-Assisted Endoscopic Retrograde Cholangiopancreatography; M-H: Mantel-Haenszel; CI: Confidence interval Studies represented the forest plot [16-20]

**Figure 5:**
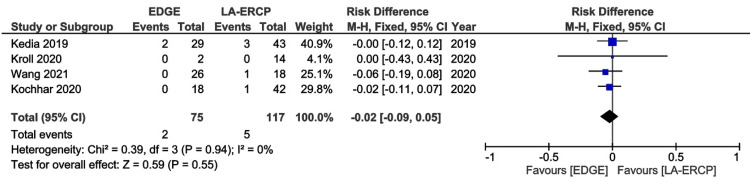
Forest Plot of Serious Adverse Events EDGE: Endoscopic Ultrasound-Directed Transgastric ERCP; LA-ERCP: Laparoscopic-Assisted Endoscopic Retrograde Cholangiopancreatography; M-H: Mantel-Haenszel; CI: Confidence interval Studies represented the forest plot [16-19]

All studies presented data concerning AEs [16-20]. The rates of AEs were also comparable between the groups, 13% in the EDGE group and 20.4% in the LA-ERCP group, with a RD - 0.08 (95% CI − 0.17 to 0.01; I² = 33%; p = 0.09) (Figure 4). A separate analysis of serious AEs was performed, including four studies [16-19]. Only 0.03% and 0.04% of these events were reported in the EDGE and LA-ERCP groups, respectively (RD -0.02: 95% CI − 0.09 to 0.05; I² = 0%; p = 0.55) (Figure 5).

Length of Hospitalization

**Figure 6:**
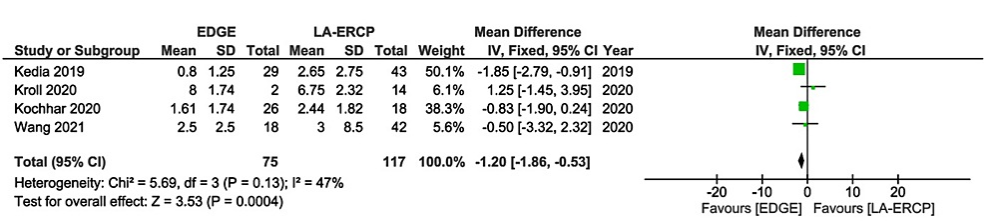
Forest Plot of Length of Hospitalization EDGE: Endoscopic Ultrasound-Directed Transgastric ERCP; LA-ERCP: Laparoscopic-Assisted Endoscopic Retrograde Cholangiopancreatography; SD: Standard deviation; IV: Inverse variance; CI: Confidence interval Studies represented the forest plot [16-19]

This analysis included four studies [16-19]. The MD of hospital stay between EDGE and LA-ERCP groups was -1.2 days (95% CI - 1.86 to - 0.53; I² = 47%; p = 0.0004), highlighting a lower LOS for the EDGE group (Figure 6).

Procedural Time

**Figure 7:**
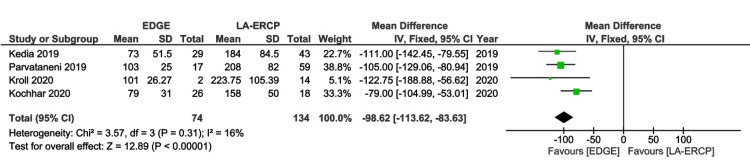
Forest Plot of Procedural Time EDGE: Endoscopic Ultrasound-Directed Transgastric ERCP; LA-ERCP: Laparoscopic-Assisted Endoscopic Retrograde Cholangiopancreatography; SD: Standard deviation; IV: Inverse variance; CI: Confidence interval Studies represented the forest plot [16-18,20]

Four studies were included in this analysis [16-18,20]. There was a significantly lower time (in minutes) in the EDGE group in comparison with the LA-ERCP group (MD - 98.62 min: 95% CI - 113.62 to - 83.63; I² = 16%; p < 0.00001) (Figure 7).

Discussion

To the best of our knowledge, this is the first systematic review and meta-analysis comparing EDGE versus LA-ERCP techniques in patients with RYGB anatomy in a dichotomic design and including only comparative studies to strengthen the quality of results. A previous meta-analysis [4]had already compared these two techniques and EA-ERCP with results consistent with ours. However, the great majority of the studies involved were not comparative, and four comparative studies included in our meta-analysis were not present in this manuscript. Although our review includes a smaller sample of patients, we prioritized quality of evidence over quantity, updated the literature search, and added different outcomes (LOS and procedural time) to the final analysis.

We decided not to include EA-ERCP in our comparison because several studies, either retrospective cohorts [21,22] or meta-analysis [3,5], had already reported lower technical and clinical success rates. These worse outcomes can be explained by some technical features inherent to the procedure, such as longer length navigated by the device causing instability, forward-viewing nature of the scope, lack of elevator mechanism, and difficulty of adequate accessories due to the narrow diameter and longer length of the working channel [2]. Thus, it is clear to us that the decision of the most appropriate approach, due to its high clinical relevance, to perform ERCP in this setting of patients rests between EDGE and LA-ERCP. However, EA-ERCP remains an acceptable option in centers where only this technique is available.

In this meta-analysis, we found similar high technical success rates and a low incidence of AEs in EDGE and LA-ERCP groups with a trend of lower AEs rates in EDGE. These results are consistent with previous non-comparative systematic reviews [23,24], demonstrating that both techniques are safe and feasible in current clinical practice. Although EDGE has the advantage of solving the clinical problem through a completely endoscopic procedure, it has the limitation of being a novel technique performed only by a select group of skilled endoscopists with therapeutic EUS training settled in tertiary hospital centers with special devices and resources. On the other hand, with the increasing availability of LAMS, there is a trend to expand its use to other transgastric procedures, enhancing its popularity among endoscopists. This context expects even higher success rates with lower AEs in this procedure.

One of the significant advantages of LA-ERCP is the possibility to perform cholecystectomy simultaneously in patients with gallbladder in situ. However, this time-consuming procedure involves a complex logistic process that demands precise time synchronization between surgical and endoscopic teams [7]. Also, it demands an operating room for execution. It has other inconveniences, such as surgical site infections, the presence of scars, longer LOS, higher costs, and the problematic performance of a consecutive ERCP if needed. For this specific context, laparoscopic-assisted intraductal exploration of the bile duct appears to be the best approach, especially if diagnosed intraoperatively [25]. Nevertheless, laparoscopic-assisted intraductal interventions are not widely performed by surgeons outside centers of excellence. On the other hand, EDGE adds the facility to allow further ERCP procedures when needed or other endoscopic interventions such as EUS-guided fine-needle aspiration/biopsy (FNA/FNB) in suspected pancreas head lesions, EUS-guided biliary drainage if traditional ERCP fails, and EUS-guided gallbladder drainage in concomitant acute cholecystitis in a non-surgical candidate for laparoscopic cholecystectomy. The aforementioned interventions are feasible since LAMS may stay in situ for days or weeks, not demanding consecutive surgical reinterventions or leaving a gastrostomy tube in situ, as in LA-ERCP, if a subsequent procedure is needed [8].

In addition, LA-ERCP may be a preferred method for urgent clinical indications demanding prompt resolution, notably cholangitis, once performed at one time. This scenario may be unfavorable for EDGE since it is well established that a two-stage procedure is preferred, with a 10-14 day interval between the stages [26,27]. This approach decreases the rate of AEs [26,27] when compared to an intermediate approach using larger (20 mm) LAMS associated with a shortened interval of two to four days between the stages and anchoring of the stent via an over-the-scope clip or endoscopic suturing [26].

The AEs in these techniques can be divided into ERCP-related and access-related complications. The first type is common for both, and the second is specific for each group. The main AE of the EDGE group in the included studies was stent dislodgement. This issue may be related to some technical procedural factors such as angulation of the LAMS and hence of the duodenoscope during ERCP, the diameter of the duodenoscope, route of LAMS placement (GG/JG), balloon dilation of the stent, fixation of the stent, the diameter of the stent, and time between stages to perform ERCP [26-29]. Retrospective cohorts [26,27] have already demonstrated the use of a 20mm diameter LAMS, the stent anchorage via an over-the-scope clip or endoscopic suturing, and the two-stage procedure execution may mitigate the risk of AEs, making this technique even safer.

In the LA-ERCP group, the AEs are mostly related to surgical access and gastrostomy step, including viscus perforation, surgical site infections (intrabdominal or superficial), and wound dehiscence. This is secondary to the high level of surgical difficulty due to adhesions and high body mass index, leading to more serious complications and open surgery conversion rates than laparoscopic cholecystectomy alone [30].

Our analysis also reported a shorter LOS and procedural time for EDGE compared to LA-ERCP. This difference was significant even for the EDGE procedures performed in two stages because, in these cases, patients were discharged after the first stage until returning for an ERCP, and all studies considered total procedure time as the sum of both stages. Even though we did not have sufficient data to compare costs, EDGE may represent a more cost-effective option than LA-ERCP due to the earlier hospital discharge and other variables, as previously demonstrated by other authors [19,31]. Overall, the high costs of LAMS could be suppressed by a decreased overall LOS and operative room costs.

Major concerns previously related to EDGE included weight regain and a high rate of fistula persistence. However, descriptive data extracted from the studies involved in this systematic review and meta-analysis did not address these concerns. Theoretically, the presence of a GG or JG fistula would turn the excluded stomach into the preferred route for food leading to weight gain. However, this is not observed in practice, as reported by several studies [8,16-19,28]. The reason for the easier closure of these created fistulas compared to the post-surgical ones rests on their pathophysiology. EDGE fistulas are created over theoretically healthy tissue with preserved histological organization and healing mechanisms [8]. In contrast, post-surgical fistulas emerge in tissue with poor vascularization and chronic inflammation, usually in an organ with downstream outlet obstruction. Additionally, no difference is reported between GG and JG closure rates [8].

The studies included in this meta-analysis demonstrated high rates of fistula closure. However, in most cases, an endoscopic technique (endoscopic suturing, over-the-scope clips, or through-the-scope clips) was used for this purpose in the same session of LAMS removal. Available data in the literature show low proportions of fistula persistence (under 10%) when no method or only argon plasma coagulation is performed at the time of LAMS removal [8,28]. On the other hand, a more recent study demonstrated a higher rate of fistula persistence (41%) after LAMS removal, suggesting that a long gap until LAMS removal, as well as a larger diameter (20mm) of the LAMS, could be associated with lower rates of fistula closure [29].

The literature recommends that LAMS should be ideally removed as soon as the pancreaticobiliary access is no longer required, with a stent dwell time of at least 14 days for fistula maturation before removal to avoid leakage into the peritoneal cavity [8,23,29]. It is also advised to assess and confirm the fistula closure through upper endoscopy or upper gastrointestinal series with barium three to four weeks after LAMS removal [8,28]. If there is persistence, endoscopic closure (with argon plasma coagulation, cap-mounted clips, and endoscopic suturing) should be attempted with satisfactory results [8,28]. However, there is still no consensus concerning the ideal time for assessing fistula persistence or if its closure should be attempted immediately after LAMS removal or only in the case of persistence. Further studies are required to determine the best approach in these situations.

This meta-analysis has limitations, for example, the high risk of bias of the studies included. This is mainly due to confounding factors and the absence of control in selecting inclusion criteria due to their retrospective design. However, no prospective cohorts or randomized controlled trials (RCTs) are available in the literature comparing both techniques, and retrospective cohorts currently represent the best quality of evidence. Even though one of the studies included was an abstract, its data did not modify the final results after the metanalysis. In addition, despite the high individual risk of bias, the evidence grade is augmented by a low heterogeneity and a low risk of publication bias amongst the articles. Another limitation is the small sample of patients included in our analysis. However, compared with the total number of procedures reported in the literature, particularly in the EDGE group, this is a significant number since it is not a procedure broadly performed. Another limitation of this systematic review is regarding the indication of the procedures once the included studies did not separate the results according to the primary disease, either biliary, pancreatic, benign, or malignant, which could have modified the outcomes.

## Conclusions

In summary, this systematic review and meta-analysis demonstrated that both approaches could be performed with similar technical success and AEs rates for this challenging population. Despite the lower procedural time and shorter hospital stay related to the endoscopic approach, the best approach should be individualized by considering personal and local experience, availability of material and devices, and specific circumstances such as the need for cholecystectomy or endoscopic reinterventions.

Therefore, EDGE and LA-ERCP are good techniques for performing ERCP in patients with RYGB, with high technical success and low AEs rates. Nonetheless, when compared to LA-ERCP, EDGE is associated with a shorter procedural time and a lower length of hospitalization. However, more prospective studies, preferably RCTs comparing these methods directly are required to provide more data about this topic to guide clinical decision-making.
